# Tranexamic acid for post-partum haemorrhage: What, who and when

**DOI:** 10.1016/j.bpobgyn.2019.04.005

**Published:** 2019-11

**Authors:** Amy Brenner, Katharine Ker, Haleema Shakur-Still, Ian Roberts

**Affiliations:** Clinical Trials Unit, Department of Population Health, London School of Hygiene and Tropical Medicine, Keppel Street, London, WC1E 7HT, United Kingdom

**Keywords:** Anti-Fibrinolytic agents, Fibrinolysis, Haemostasis, Maternal health, Postpartum haemorrhage, Tranexamic acid

## Abstract

Tranexamic acid reduces bleeding by inhibiting the breakdown of blood clots. It is cost-effective and heat-stable with a long shelf life. In the WOMAN trial, tranexamic acid reduced deaths due to bleeding with no increase in thromboembolic events. The effect was greatest when women received tranexamic acid within 3 h of childbirth (RR = 0.69, 95% CI 0.52–0.91). The WHO recommends that women with post-partum haemorrhage receive 1 g tranexamic acid intravenously as soon as possible after giving birth, followed by a second dose if bleeding continues after 30 min or restarts within 24 h since the first dose. Urgent treatment is critical because women with post-partum haemorrhage bleed to death quickly, and tranexamic acid is most effective when given early. Evidence suggests there is no benefit when the drug is given more than 3 h after bleeding onset. Alternative routes of administration and use of tranexamic acid in the prevention of post-partum haemorrhage are research priorities.

## Highlights

• Tranexamic acid reduces bleeding by inhibiting the enzymatic breakdown of fibrin.
• It is the only treatment proven to reduce deaths due to haemorrhage in a randomised trial.
• It is a safe, effective and affordable treatment for post-partum haemorrhage.
• Early administration reduces the risk of exsanguination by one-third.
• Urgent treatment is critical: treat as soon as possible and not >3 h after birth.

## Background

The anti-fibrinolytic drug tranexamic acid was invented by husband and wife research team Shosuke and Utako Okamoto working at Keio and Kobe Medical Schools in Japan in the 1950s and early 1960s. Their objective was to identify a drug that would reduce maternal death from post-partum haemorrhage. In 1950, Japan had a maternal mortality ratio of approximately 180 deaths per 100,000 live births, which is similar to that currently found in some low- and middle-income countries. A large proportion of these maternal deaths were due to post-partum haemorrhage. The couple were aware that the fibrinolytic enzyme plasmin worsened bleeding by increasing blood clot breakdown, and hence, they sought to determine an effective antiplasmin. They began their search by studying epsilon-aminocaproic acid (EACA), which is now a widely used antifibrinolytic agent. However, they extended their research for a more potent drug, and in 1962, writing in the Keio Journal of Medicine, they reported the invention of 1-(amino methyl)-cyclohexane-4-carboxylic acid (AMCHA), now known as tranexamic acid, a chemical relative of EACA but 27 times more powerful [1]. Tranexamic acid is a synthetic analogue of the amino acid lysine. It can be administered orally or by a short intravenous infusion, after which peak plasma concentrations of tranexamic acid are obtained rapidly. It is excreted as an unchanged drug in the urine with an elimination half-life of approximately 3 h.

## Tranexamic acid and fibrinolysis

Tranexamic acid prevents bleeding by inhibiting the enzymatic breakdown of fibrin blood clots. Fibrin breakdown starts when the glycoprotein pro-enzyme plasminogen, which is produced by the liver, is converted into the fibrinolytic enzyme plasmin by tissue plasminogen activator (tPA). The plasminogen protein is folded into a number of molecular loops called kringles that stick out like fingers. Plasminogen binds to fibrin through the lysine-binding sites at the tips of these ‘fingers’ [2]. If the lysine residues on fibrin are removed, the binding of plasminogen is inhibited [3]. tPA is released from the vascular endothelium in response to tissue damage, ischaemia and the presence of thrombin. tPA also binds to fibrin through the lysine-binding sites. Fibrin binds both plasminogen and tPA, thus localising plasmin formation. Plasmin bound to fibrin is protected from plasmin inhibitors. Plasmin splits the fibrin blood clot into fibrin degradation products (FDPs). This process exposes more lysine residues, which further bind more plasminogen, thus accelerating fibrinolysis in a positive feedback loop. Tranexamic acid is a molecular analogue of lysine that inhibits fibrinolysis by reducing the binding of plasminogen and tPA to fibrin.

## Tranexamic acid and surgical bleeding

Bleeding is a major complication of surgery. Severe surgical bleeding is associated with increased morbidity and mortality, as well as increased blood transfusion and longer duration of hospital stay [4]. Fibrinolytic activity is elevated during and after surgery following the release of tPA. For several decades, tranexamic acid has been used to reduce surgical bleeding in some patients undergoing various types of surgery including orthopaedic; cardiac; cranial; hepatic; ear, nose and throat and gynaecological operations. A systematic review and meta-analysis of 129 trials that included 10,488 patients found that tranexamic acid reduced the risk of blood transfusion by over one-third (RR = 0.62, 95% CI 0.58–0.65; p < 0.001) [5]. This effect remained when the analysis was restricted to trials with adequate allocation concealment and blinding.

## Tranexamic acid and traumatic bleeding

The evidence that tranexamic acid significantly reduces surgical bleeding raised the possibility that it might also reduce traumatic bleeding, which is a leading cause of potentially preventable mortality in trauma patients, and this led to the initiation of the CRASH-2 (Clinical Randomisation of Anti-fibrinolytic in Significant Haemorrhage) trial in 2005. The CRASH-2 trial was a large multicentre randomised trial of the effect of tranexamic acid on death and vascular occlusive events in patients with bleeding trauma. The results were published in 2010 [6]. A total of 20,211 adult patients with trauma along with significant bleeding, who were within 8 h of their injury, were randomly allocated to receive tranexamic acid (1 g over 10 min, followed by an infusion of 1 g over 8 h) or matched placebo. The primary outcome was death in hospital within 4 weeks. Tranexamic acid significantly reduced death due to bleeding (RR = 0.85, 95% CI 0.76–0.96) and all-cause mortality (RR = 0.91, 95% CI 0.85–0.97), with no increase in vascular occlusive events. The reduction in death due to bleeding was greatest when tranexamic acid was given within 3 h of injury (RR = 0.72, 95% CI 0.63–0.83). When it was given beyond 3 h of the injury, there was no mortality benefit, and some suggest an increased risk of bleeding, possibly a manifestation of thrombotic disseminated intravascular coagulation. Pre-specified subgroup analyses showed that early administration of tranexamic acid reduced death from bleeding regardless of the type of injury (both blunt and penetrating trauma) and bleeding severity (as judged by baseline systolic blood pressure and Glasgow Coma Score) [7]. On the basis of the results of the CRASH-2 trial, tranexamic acid was included on the World Health Organization's (WHO) List of Essential Medicines and was also incorporated into trauma protocols in many countries around the world. Using data from the CRASH-2 trial, a cost-effectiveness analysis concluded that the early treatment of patients with bleeding trauma with tranexamic acid is likely to be highly cost-effective in low-, middle- and high-income countries [8].

## Tranexamic acid and post-partum bleeding

Post-partum haemorrhage is the leading cause of maternal mortality worldwide. There were approximately 300,000 maternal deaths worldwide in 2010, over a quarter (27%) of which were estimated to be due to haemorrhage [9]. This means that there is one maternal death from post-partum haemorrhage every 6 min somewhere in the world. Although most of the deaths occur in low- and middle-income countries, post-partum haemorrhage is also a leading cause of maternal mortality in high-income countries. Women with post-partum haemorrhage have elevated levels of FDPs including D-dimers, a biomarker of fibrinolysis [10], [11]. Although existing medical and surgical interventions can be used to treat post-partum bleeding, tranexamic acid offers an alternative way to support haemostasis by inhibiting the enzymatic action of plasmin on fibrin. Given that tranexamic acid reduces surgical bleeding, it clearly had the potential to improve outcomes for women with post-partum haemorrhage.

## The WOMAN trial

Although there were several small trials of tranexamic acid in obstetric bleeding, most of which showed a decrease in blood loss, they were of poor quality and too small to assess important maternal outcomes. Whilst the CRASH-2 trial was still underway, the WOMAN (World Maternal Antifibrinolytic) trial was launched in 2009, with an aim to provide robust, definitive evidence on the use of tranexamic acid in post-partum haemorrhage. The results of this multicentre, randomised, double-blind, placebo-controlled trial of the effect of tranexamic acid on death or hysterectomy in women with post-partum haemorrhage were published in 2017 [12]. A total of 20,060 women with a clinical diagnosis of post-partum haemorrhage were randomly allocated to receive tranexamic acid (1 g intravenously followed by a second dose if bleeding continued after 30 min or restarted within 24 h of the first dose) or matched placebo. The primary outcome was death from all causes or hysterectomy within 42 days of randomisation, with death due to bleeding as the key secondary outcome. Based on the results of the CRASH-2 trial [7] and temporal changes in fibrinolysis observed after childbirth [13], a pre-specified subgroup analysis of the effect of tranexamic acid on death due to bleeding by time to treatment was conducted [14]. Other subgroup analyses included the effect of tranexamic acid on death due to bleeding by type of delivery and cause of haemorrhage.

Tranexamic acid significantly reduced death due to bleeding (RR = 0.81, 95% CI 0.65–1.00; p = 0.045), with no increase in thromboembolic events or complications. The effect on death due to bleeding was greatest when tranexamic acid was given within 3 h of childbirth, (RR = 0.69, 95% CI 0.52–0.91; p = 0.008). When it was given beyond 3 h of childbirth, there was no apparent reduction in death due to bleeding (RR = 1.07, 95% CI 0.76–1.51; p = 0.70). There was no evidence that the treatment effect varied by type of delivery or cause of haemorrhage.

While the trial was underway, it became apparent that the decision to perform a hysterectomy was often made at or before randomisation. It was thought that the inclusion of such procedures and non-bleeding causes of death in the analysis would dilute the treatment effect [14]. As expected, there was no effect on the composite outcome of death from all causes or hysterectomy. In the WOMAN trial, and other trials of haemostatic treatments, death due to bleeding was the most appropriate endpoint. Twenty-eight per cent of deaths in the WOMAN trial were attributed to non-bleeding causes including complications such as sepsis and organ failure, which are unlikely to be affected by tranexamic acid. The treatment effect on all-cause mortality is diluted towards null because it is a weighted average of the cause-specific effects. Sample size depends inversely on the square of the effect size; hence, death due to bleeding has greater statistical power than all-cause mortality, especially when non-bleeding deaths are common [15]. Furthermore, all-cause mortality is not generalisable because the relative contribution of different causes of death varies between populations, and any harmful treatment effects may be obscured [16].

A sub-study within the WOMAN trial examined the mechanism of action of tranexamic acid. One hundred sixty-seven participants in the WOMAN trial in Nigeria were included in the ETAC (Effect of Tranexamic Acid on Coagulation) study, which aimed to assess the effect of tranexamic acid on fibrinolysis and coagulation during post-partum haemorrhage by comparing D-dimer levels, clot lysis and coagulation in the treatment and placebo groups [17]. The study found that increased fibrinolysis is common is women with post-partum haemorrhage and that fibrinolysis is reduced with tranexamic acid, as demonstrated by lower D-dimer levels in the tranexamic acid group (−2.16 mg/L, 95% CI −4.31 to 0.00, p = 0.05) [18].

## Use of tranexamic acid in a clinical setting

Tranexamic acid should be readily available at all times in emergency obstetric care facilities: it is cost-effective, heat stable and widely available, with a long shelf life. An economic evaluation that used data from the WOMAN trial to assess the cost-effectiveness of early tranexamic acid for usual care of women with post-partum haemorrhage in Nigeria and Pakistan concluded that it is likely to be highly cost-effective [19]. A Cochrane review of anti-fibrinolytic agents used for the treatment for post-partum haemorrhage identified three eligible trials, two of which compared intravenous tranexamic acid with placebo or standard care (the WOMAN trial and a French trial) [20]. The French trial randomly allocated 152 women with post-partum haemorrhage, defined as blood loss >800 mL following vaginal delivery, to receive high-dose tranexamic acid (a loading dose of 4 g over 1 h followed by an infusion of 1 g over 6 h) or standard care [21]. A meta-analysis of 20,172 women from the WOMAN trial and the aforementioned French trial showed that tranexamic acid reduces the risk of death due to bleeding (RR = 0.81, 95% CI 0.65–1.00), with early treatment being more effective. On the basis of this review, the WHO updated its recommendation on the use of tranexamic acid for the treatment of post-partum haemorrhage [22]. The WHO strongly recommends early treatment of post-partum haemorrhage (within 3 h of birth) with intravenous tranexamic acid using the same dosing regimen as that used in the WOMAN trial – a fixed dose of 1 g in 10 mL (100 mg/mL) intravenously at a rate of 1 mL per minute. A second 1 g intravenous dose should be administered if bleeding continues after 30 min or restarts within 24 h of the first dose. Tranexamic acid should be given to all women with ‘clinically estimated blood loss of more than 500 mL after vaginal birth or 1000 mL after caesarean section, or any blood loss that is sufficient to compromise haemodynamic stability’, regardless of the cause of haemorrhage [22].

Tranexamic acid should be used in addition to all usual treatments for the management of post-partum haemorrhage including medical (uterotonics), non-surgical and surgical interventions. Although it can be mixed with some solutions for infusion including electrolyte solutions, carbohydrate solutions, amino acid solutions and dextran solutions, it should not be mixed with blood for transfusion or solutions containing mannitol or penicillin. Other restrictions of use include women with a clear contraindication to anti-fibrinolytic therapy such as a known thromboembolic event during pregnancy, known hypersensitivity to tranexamic acid, active intravascular clotting or a history of coagulopathy.

## Why urgent treatment is critical

**Fig. 1:**
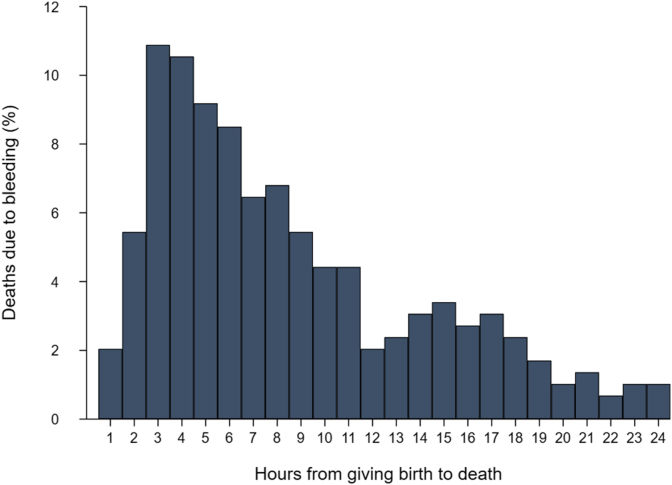
Distribution of deaths due to bleeding by hours since birth in the WOMAN trial.

Time is of essence with regard to treating patients with life-threatening bleeding. Urgent treatment of post-partum haemorrhage with tranexamic acid is important for two reasons. First, women with post-partum haemorrhage bleed to death quickly. Most deaths due to haemorrhage occur soon after childbirth, with more than half occurring within 8 h (see Fig. 1). If treatment is delayed, many women who could have benefited will have exsanguinated. Second, tranexamic acid is most effective when given early. In the WOMAN trial, a pre-specified subgroup analysis found that early treatment within 3 h of childbirth reduced the risk of death due to bleeding by nearly one-third (RR = 0.69, 95% CI 0.52–0.91; p = 0.008). An individual patient data meta-analysis of the WOMAN and CRASH-2 trials showed that there was a 10% reduction in survival benefit for every 15-min delay in treatment with tranexamic acid [23]. Not only is earlier treatment more beneficial, but evidence also suggests that tranexamic acid has no effect when given more than 3 h after bleeding onset, and late treatment may even be harmful. The updated WHO recommendation on the use of tranexamic acid for post-partum haemorrhage emphasises the importance of giving tranexamic acid as soon as possible and no more than 3 h after childbirth [22].

## Tranexamic acid and other maternal outcomes

If, as recommended by the WHO, tranexamic acid is used as a first-line treatment for post-partum haemorrhage, its effects on other patient outcomes could potentially exceed those observed in the WOMAN trial. Because post-partum haemorrhage is a medical emergency, interventions to resuscitate the woman and control her bleeding inevitably take priority over randomisation into a trial. The use of such interventions is often dictated by major haemorrhage protocols [24]. Presenting signs and symptoms of hypovolaemia dependent on the extent of pre-randomisation blood loss will often determine treatment. Blood transfusion and surgical interventions for bleeding such as hysterectomy therefore have limited utility as outcome measures, as they lack the potential to be influenced by the trial treatment [16]. Indeed, we observed no effect on transfusion (RR = 1.00, 95% CI 0.98–1.03; p = 0.86) or hysterectomy (RR = 1.02, 95% CI 0.88–1.07; p = 0·84) in the WOMAN trial [12]. Similarly, there was no effect on transfusion in the CRASH-2 trial [6]. In contrast, tranexamic acid reduces transfusion in surgical bleeding [5]. This discrepancy may have arisen because tranexamic acid can be given both before and during surgery to reduce intraoperative and postoperative bleeding, whereas in an emergency setting, it is given to treat existing bleeding at the same time as other interventions for resuscitation and haemorrhage control. In an exploratory analysis of the WOMAN trial dataset, when early hysterectomies assumed to have been planned at or before randomisation were excluded, there was a non-significant reduction in hysterectomy for bleeding (RR = 0.65, 99% CI 0.39–1.08) [25]. Although imprecise, this effect has a similar magnitude as that of the reduction in laparotomy for bleeding in the main analysis (RR = 0.64, 95% CI 0.49–0.85) [12].

## What next?

Tranexamic acid is a safe, effective and affordable treatment for post-partum haemorrhage. The current research agenda must now address the need for interventions to prevent post-partum haemorrhage, particularly in high-risk groups. In 2015 and 2016, two systematic reviews identified 12 and 26 trials of tranexamic acid for the prevention of post-partum haemorrhage, respectively ∗[26], [27]. The included studies were generally small and unreliable with some serious flaws and therefore provided insufficient evidence. Since then, the results of the TRAAP (Tranexamic Acid for Preventing Postpartum Haemorrhage Following a Vaginal Delivery) trial, a multicentre, placebo-controlled, double-blind trial that randomised 4079 women to receive either tranexamic acid or placebo, have been published. There was no reduction in post-partum haemorrhage when defined as blood loss of at least 500 mL; however, there was a 25% reduction in clinically significant post-partum haemorrhage [28]. These findings suggest tranexamic acid has the potential as a prophylactic, but a larger trial is needed to confirm this. The WOMAN-2 trial will assess the effectiveness of tranexamic acid for the prevention of post-partum haemorrhage in women with moderate or severe anaemia, which confers a considerably increased risk [29], [30].

In high-income countries, most women deliver in hospital or have access to ambulance transportation; hence, doctors or paramedics can administer intravenous tranexamic acid to those with post-partum haemorrhage. In low- and middle-income countries, approximately 40% of women deliver at home with only rudimentary transport. Although health workers attend most births, most cannot give intravenous drugs. Transport to hospital can take hours, and many women exsanguinate before arrival. Although intravenous tranexamic acid is the treatment of choice, this is not an option for tens of thousands of women. Intramuscular tranexamic acid could increase timely access to effective care. In healthy volunteers, intramuscular tranexamic acid achieves therapeutic levels (>10 mg/L) at approximately 30 min. Health workers are trained to give intramuscular oxytocin and could give intramuscular tranexamic acid if shown to be effective. Intramuscular use could be a lifesaving treatment in the community; therefore, more women can be treated and sooner. Tranexamic acid has a wide therapeutic index, and a further intravenous injection could be given if and when this becomes possible. Alternative routes of administration of tranexamic acid should be a research priority, as recommended by the WHO [22].

Owing to improvements in emergency obstetric care, including use of tranexamic acid as a first-line therapy, more women will survive a post-partum haemorrhage than ever before. Furthermore, the incidence of post-partum haemorrhage is increasing [31], [32], [33]. Consequently, the number of women who proceed to experience physical and psychological consequences of post-partum haemorrhage will also increase. More research is needed on the risk factors for maternal morbidity after post-partum haemorrhage and on the ways to reduce it [34].

## Summary

Tranexamic acid is a molecular analogue of lysine that inhibits fibrinolysis – the enzymatic breakdown of fibrin blood clots – by reducing the binding of plasminogen and tPA to fibrin, thereby preventing bleeding. The WOMAN trial demonstrated that tranexamic acid is a safe and effective treatment for post-partum haemorrhage. When given early, tranexamic acid reduces deaths due to bleeding by one-third. Urgent treatment is critical because women with post-partum haemorrhage bleed to death quickly, and tranexamic acid is most effective when given within 3 h of childbirth, with no apparent benefit thereafter. Following the results of the WOMAN trial, the WHO recommend that women with clinically diagnosed post-partum haemorrhage receive 1 g of tranexamic acid intravenously as soon as possible and no more than 3 h after childbirth, followed by a second dose if bleeding continues after 30 min or restarts within 24 h of the first dose. Research priorities include alternative routes of administration, tranexamic acid use for the prevention of post-partum haemorrhage and risk factors of morbidity after post-partum haemorrhage.

**Practice points**

• Treat women with clinically diagnosed post-partum haemorrhage, defined as clinically estimated blood loss of more than 500 mL after vaginal birth or 1000 mL after caesarean section, or any blood loss that is sufficient to compromise haemodynamic stability.
• Women with post-partum haemorrhage should receive a fixed dose of 1 g tranexamic acid in 10 mL (100 mg/mL) intravenously (1 mL per minute) as soon as possible after delivery and no more than 3 h after birth.
• A second dose of 1 g should be given intravenously if bleeding continues after 30 min or restarts within 24 h of the first dose.
• Tranexamic acid should be given in addition to usual treatments for the management of post-partum haemorrhage including medical (uterotonics), non-surgical and surgical interventions, regardless of the cause of haemorrhage or the mode of delivery.
• Tranexamic acid should not be mixed with blood for transfusion or solutions containing mannitol or penicillin.

**Research agenda**

• Tranexamic acid use for the prevention of post-partum haemorrhage, particularly in high-risk groups.
• Alternative routes of administration of tranexamic acid to increase accessibility and reduce time to treatment.
• Studies to examine the risk factors of maternal morbidity after post-partum haemorrhage and to investigate possible ways to reduce these risk factors.

## Conflicts of interest

None.

## Funding

Funding for the WOMAN trial was provided by London School of Hygiene & Tropical Medicine, Pfizer (for the trial drug and placebo), the UK Department of Health (grant number HICF-T2–0510-007), the Wellcome Trust (grant number WT094947), and The Bill & Melinda Gates Foundation (grant number OPP1095618).
